# Systems solutions by lactic acid bacteria: from paradigms to practice

**DOI:** 10.1186/1475-2859-10-S1-S2

**Published:** 2011-08-30

**Authors:** Willem M de Vos

**Affiliations:** 1 Laboratory of Microbiology, Wageningen University, The Netherlands, Department of Veterinary Biosciences, and Department of Bacteriology & Immunology, Helsinki University, Finland

## Abstract

Lactic acid bacteria are among the powerhouses of the food industry, colonize the surfaces of plants and animals, and contribute to our health and well-being. The genomic characterization of LAB has rocketed and presently over 100 complete or nearly complete genomes are available, many of which serve as scientific paradigms. Moreover, functional and comparative metagenomic studies are taking off and provide a wealth of insight in the activity of lactic acid bacteria used in a variety of applications, ranging from starters in complex fermentations to their marketing as probiotics. In this new era of high throughput analysis, biology has become big science. Hence, there is a need to systematically store the generated information, apply this in an intelligent way, and provide modalities for constructing self-learning systems that can be used for future improvements. This review addresses these systems solutions with a state of the art overview of the present paradigms that relate to the use of lactic acid bacteria in industrial applications. Moreover, an outlook is presented of the future developments that include the transition into practice as well as the use of lactic acid bacteria in synthetic biology and other next generation applications.

## Introduction

The historic use of bacteria that produce lactic acid and collectively are designated lactic acid bacteria (LAB) is well documented for a variety of food fermentations, some even dating back to the earliest written records. However, less exposed is the impact of LAB in the diet of our far-away ancestors that lived over a million of years ago. There is considerable support for the hypothesis that lactobacilli and other notably plant-related LAB have been consumed in large amounts in neolithic times [1]. This so called paleo-diet may have contained over a million more microbes than our present foods, resulting in a high and continuous load of LAB. In retrospect, this provides an explanation why some LAB have developed intimate interactions with our body and several LAB are successfully marketed as probiotics [2,3].

Traditionally LAB have been considered to include low G+C content Gram-positive bacteria included in the phylum Firmicutes that are used as starters for industrial food fermentations, notably those based on raw materials derived from milk, meat and plants. These fermentations together with probiotic products represent a total global market value of over 100 Billion Euro (Table 1) [3,4]. Economically by far the most important products derive from industrial dairy fermentations and include cheese, yoghurt and other fresh dairy produce. These fermentations are initiated by well-known genera of LAB that include *Lactobacillus*, *Lactococcus* and *Streptococcus*. However, the market for probiotic bacteria in foods and supplements is the most rapidly growing segment in the fast moving consumer goods and expected to grow by 10 % each year [3]. Most of the applied probiotic bacteria are *Lactobacillus* spp. However, approximately one third are Bifidobacteria, a group of bacteria with a high G+C content included in the phylum Actinobacteria that also produce lactic acid but always in combination with acetic acid. Bifidobacteria are almost exclusively found in association with animal hosts [5]. Hence, it is no surprise that some *Bifidobacterium* strains are also marketed as probiotic bacteria [2,3]. As various reviews on the genomics and metabolism of Bifidobacteria have been reported recently [5-7], specific attention for this group is not in the remit of this paper that will focus on the true LAB.

**Table 1:** Economic value of fermentations including LAB and Bifidobacteria. Data taken from recent market reviews and estimations [3,4].

Product	Global Market Value (Euro)	Main Bacterial Genera
Cheese Products	55 Billion	*Lactococcus* &*Lactobacillus*
Yoghurt & Fresh Dairy	25 Billion	*Streptococcus* &*Lactobacillus*
Probiotic Products	20 Billion	*Lactobacillus* &*Bifidobacterium*

**Figure 1:**
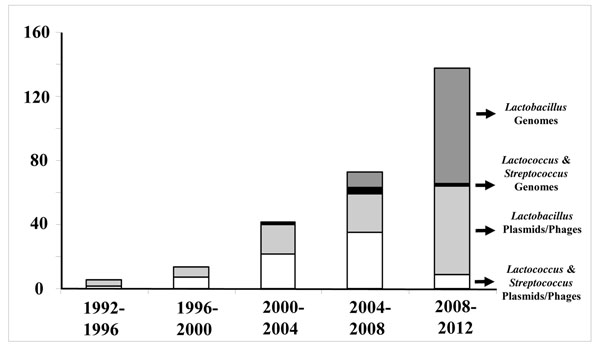
Development of genome sequencing in the last two decades. The number of entries in the NCBI database were scored and summed for the plasmids and bacteriophages [phages] as well as the genomes from *Lactobacillus* or *Lactococcus* and *Streptrococcus* spp. that are considered as LAB.

At the time that we are celebrating the 30 year anniversary of the LAB symposia with the present LAB10, it is appropriate to reflect and benchmark the position where the science in this area has led us. Even more important is the possibility to look ahead and present some future perspectives in this area. This is done here with a focus on the practical impact of the paradigm bacteria that underpin a multibillion Euro industry (Table 1). Moreover, this is guided by the three major developments that have brought the research on these powerhouses of the probiotic and dairy industry at the level that it has now. The first is the genomic revolution that has been quickly implemented in LAB with the sequence analysis of plamids, bacteriophages, and now genomes or collections of genomes (Fig. 1). The second is the high throughput experimentation that has become available and shown to be of particular use for LAB. The final development relates to the systems and synthetic biology approaches. These systems solutions integrate all aspects of the metabolism, genetics and application of LAB that have been the leading theme of the past LAB symposia leading to the present LAB10.

## Paradigm genetic elements – early industrial impact

An important part of the research on LAB is characterized by a strong focus on molecular biology and genetics. This reflects the attention for this discipline originating with the development of biotechnology. However, another relevant factor contributing to the attention for the genetics of LAB is the large impact of genetic elements, such as plasmids and bacteriophages. It appeared from the pioneering work of Larry L. McKay that lactococci, then known as lactic streptococci, harbor large plasmid complements and code for important functions [8]. Many of these plasmids and bacteriophages have been characterized at the sequence level and deposited in the NCBI database accounting for a large genomic collection in the early nineties (Fig. 1). However, even prior to this time, when deposition was not common, many lactococcal plasmids have been sequenced, starting more than 25 years ago with the 2.2 kb pSH71 replicon [9,10]. Many mobile elements that also include conjugative transposons were found to encode important industrial characteristics such as lactose and citrate metabolism, proteinase, bacteriocin and exopolysaccharide (EPS) production, as well as bacteriophage insensitivity. The early discoveries that originate from a basic characterization of their molecular properties have resulted in a variety of applications and these paradigms are summarized here (Table 2).

**Table 2:** Paradigms of the mobile and other genetic elements in LAB.

Function	Paradigm Element [Genes]	Discovery or Exploitations	References
Replication	pWV01/pSH71 [*repAB*]	Plasmid Vectors and Copy Number	[9-11]
Lactose Metabolism	pLP712 [*lacFEG*]	Controlled Expression; Food Grade Markers	[12-14]
High Frequency Conjugation	pLP712 [*cluA*]	Clumping and High Frequency Conjugal Transfer	[15]
Proteinase Production	pWV05 – pSK112 [*prtPM*]	Chaperon Function; Flavor Engineering	[16-18]
Citrate Metabolism	pCT176 [*citP*]	Citrate Transport; Flavor Engineering	[19]
Bacteriophage Resistance	pTR2030 [*abiA*-*abiZ*]	Bacteriophage Resistant Starters	[20-22]
Nisin and Phage Resistance	pNP40	Bacteriophage Resistance Starters	[23]
Sucrose-Nisin Transposon	Tn5276 [*sac* &*nis* operons]	Conjugal Transfer; Antimicrobial Strains; NICE system]	[24,26]
EPS Production	pNZ4000 [*eps* operon]	EPS Priming Polymerase; Structure Engineering	[27]
Temperate Phage	Bacteriophage r1t	Temperature Controlled Expression	[28-30]
Mucus Binding	GGISL2 [*spaCBA*-*srtA*]	Genomic Island for Mucus Binding Pili	[31]
Phage and Plasmid Resistance	CRISPR-*cas* operon	Bacteriophage & Plasmid Immunity System	[32-34]
Production of SLP	SLP	Phase Variation and SLP Induced DC SIGN signalling	[35-37]

The canonical functions of the elements are provided with its genetic designation and, if appropriate, the relevant genes. Moreover, the potential impact of elements is indicated with key references. The elements include plasmids, transposons, genomic islands, gene cassettes or other discrete genomic sequences. NICE: Nisin Inducible Controlled Expression, SLP; Surface Layer Protein.

Among the most important applications with significant industrial impact has been the stacking of genetic elements in starter cultures that has been exploited to great extent to increase bacteriophage resistance. As the improved strains are obtained via natural conjugation systems, no genetically modified organisms (GMOs) are generated, and hence this has become one of the hallmarks of industrial strain improvement of LAB [21,22,38]. The number of bacteriophage insensitivity systems is ever increasing and these continue to generate practically useful strains, specifically for the dairy practice where an enormous load of bacteriophage is encountered. Similarly, conjugative transposons encoding metabolic properties, such as the sucrose-nisin transposon and derivatives have been discovered [24]. These have been exploited for generating non-GMO starter strains that produce nisin and hence reduce contaminating *Listeria* and other pathogens [39]. Moreover, new genetic elements continue to be discovered by comparative and functional genomics approaches. These include the conjugative maltose transposon detected in a plant-derived strain of *Lactococcus lactis* and transferable to model strains of *Lactococcus lactis*[40]. In addition, genetic islands have been discovered and found to encode the production of mucus-binding pili in the probiotic *Lactobacillus rhamnosus* GG [31]. Finally, a highly important system that protects prokaryotes from invasion by foreign DNA such as bacteriophages and plasmids, is the CRISPR system that has been discovered in *Streptococcus thermophilus*[*32*-*34*]. About half of the bacteria contain this CRISPR system and this also applies to the LAB where, for instance, it is absent in *Lactobacillus plantarum* and hence it can be considered as a specific genetic element. The CRISP system forms a nucleic acid based immunity system requiring the action of the specific endonuclease activity of the CRISPR-associated Cascade complex that recently has been worked out in detail [41]. The CRISPR sequences are identical to parts of the bacteriophage genomes to which the strains are immune and hence they have significant diagnostic value as they are indicative of the bacteriophage history [34]. Moreover, the CRISPR-system forms an important basis for the rational production of bacteriophage-insensitive mutants that are essential for successful industrial fermentations. It was observed that the Cascade system can be functionally transferred [42]. This opens avenues for its further exploitation in LAB that do not contain it naturally.

Several of the plasmid replicons such as that from the related plasmids pSH71 and pWV01, have been studied extensively and formed the basis for optimizing host-vector and transformation systems [10,11,43]. It is of interest to note that many of the gene cloning, expression and secretion systems developed more than 25 years ago [at the time that lactococci were still known as streptococci], are still in use today in only slightly modified form [9,11]. This also holds for food-grade cloning and expression systems that were developed based on extensive molecular characterization of the lactococcal lactose metabolism [12,14]. Moreover, detailed analysis of the nisin biosynthetic pathway led to the discovery of peptide-mediated communication modules [25]. These formed the basis for the Nisin Induced Controlled Expression (NICE) systems that are used worldwide for controlled gene expression [26,44]. The utility of this system was demonstrated in dozens of studies [45]. An important spin off was the development of controlled lysis in cheese starters that may contribute to accelerated cheese ripening [46]. Similarly, the genomic characterization of the first complete lactococcal bacteriophage resulted in the development of a controlled expression system based on its regulatory circuit [29,30].

## Paradigm strains of LAB – from genetic models to application

Given the interest and developments in the LAB genetics it is no surprise that genomics approaches were embraced in the early days (Fig. 1). As complete genome sequencing was relative slow and notably expensive, there was a strong focus on a limited set of model strains that have developed into paradigms for further applications. The early LAB research was dominated by attention for *Lactococcus lactis* due its importance as starter culture for industrial dairy fermentations (Table 1). As the dairy lactococci contain a large plasmid complement, plasmid-free strains were developed and two elegantly constructed strains MG1363 and IL1403 were developed in the early 80’s [47,48]. In retrospect these strains served as models for *Lactococcus lactis* subsp. *cremoris* (MG1363) and *lactis* (IL1403). A draft genome of the 2.4 Mb *Lactococcus lactis* IL1403 was generated ten years ago [49] and finalized [50], whereas that of the strain MG1363 with a slightly larger size of 2.5 Mb was reported somewhat later [51]. As MG1363 is by far the most widely used lactococcal model strain, its genome was recently resequenced, resulting in the correction of multiple mistakes [52]. The latter study also provided the complete genome sequence of the derivative strain NZ9800, carrying Tn*5276* and a 4-bp deletion in the *nisA* gene, which is used as a host for the NICE system [44]. These paradigm strains of *Lactococcus lactis* form the basis for hundreds of genetic and metabolic studies that have been carried out today. Remarkably, new series of discoveries continue to be made as has recently been shown by the presence of a pellicle polysaccharide that covers the surface of strain MG1363 that [53].

The notion that Lactobacilli are of great importance specifically for probiotic culture developments has greatly stimulated their genomic characterization. The genome of *Lactobacillus plantarum* was the first to be completed and found to be among the largest genomes to date with a size of 3.3 Mb [54]. This organism was selected as it was a human isolate, was able to grow fast on a variety of sugars, and was accessible to high efficiency genetic transformation. Moreover, it had been found to efficiently survive the intestinal tract passage making it an ideal paradigm probiotic or delivery strain [55]. The genetic blue print of *Lactobacillus plantarum* served as a basis for rapid insight into its use in food fermentations as well as a probiotic microbe and sparkled a wave of research interest generating over 100 publications that are related to its genome. The hallmark discoveries related to its genome include the finding that the degree of alanylation of its lipotechoic acids (LTAs) affects its immune response [56], the notion that its survival in the human and murine host induces over 500 genes that are not expressed in laboratory media [57], and the observation that its exposure in the upper intestinal tract of human induces a marked anti-inflammatory immune response [58]. Moreover, *Lactobacillus plantarum* has served to construct the first genome-based metabolic model generated for LAB [59] and sparkled may other metabolic, genetic and immunological studies that have recently been reviewed [60-62].

While *Lactococcus lactis* and *Lactobacillus plantarum* have developed into true paradigm strains, many other LAB are attractive candidates for model studies as they have unique features and have large economic impact (Table 1). A selection of these are summarized here with attention for the genomic characterization and impact in research and application (Table 3). Many of them are highly transformable and this has promoted their use as genetic model systems. It is of interest to note that there is an inverse correlation between the presence of the CRISPR – cas system [34] and transformability and in retrospect it explains the relatively late discovery of this important immunity system in LAB (Table 3). Important probiotic paradigms are *Lactobacillus acidophilus* NCFM and *Lactobacillus rhamnosus* GG, both of which are worldwide used as probiotic strains [2]. Their genomes have sparked the discovery of probiotic mechanisms, some of which are located on special genetic elements [Table 2]. These include the SLP-element that is affected by induced phase variation in *Lactobacillus acidophilus* NCFM and produces SlpA that mediates signaling to the DC-SIGN receptor of dendritic cells [37]. In addition, the genomic island ISSL1 in *Lactobacillus rhamnosus* GG was found to encode the production of pili, protruding filaments of around a micron in length that so far only have been discovered in Gram-positive pathogens [31]. These pili were decorated with the pilus protein SpaC that bound to human mucus, providing a molecular basis for competitive exclusion with other mucus-binding pathogens [[31], and unpublished observations]. It was observed earlier that reducing the degree of alanylation, and hence the positive charge, of LTA in *Lactobacillus plantarum* affected its immune stimulation and reduced colitis in a murine model [56]. Similarly, it was recently reported that the complete removal of LTAs acids in *Lactobacillus acidophilus* induced its anti-inflammatory signaling to dendritic cells and also reduced colitis in a murine model [67]. These observations provide a molecular explanation for previous findings for the in vitro interaction between lactobacilli and the immune system. This was recently confirmed in healthy volunteers where a specific immune response of the upper intestinal tract was observed following exposure to cells of *Lactobacillus plantarum*[58] and other probiotic strains, including *Lactobacillus rhamnosus* GG and a *Lactobacillus acidophilus* strain [68]. Another seminal finding derived from the genomic characterization of the probiotic strain *Lactobacillus salivarius* UCC118 and the detection of the coding capacity of a broad-spectrum class II bacteriocin that inactivated *Listeria monocytogenes*[66] Wild-type *Lactobacillus salivarius* UCC118 but not its bacteriocin-negative mutant was found to protect mice from the killing effect of challenges of *Listeria monocytogenes*, illustrating another probiotic mechanisms [69].

With increasing technological developments and reduction of sequencing costs, more genomes were sequenced in the last decade, including those of the yoghurt strains *Lactobacillus bulgaricus*[64] and *Streptococcus thermophilus*[65]. An important development was the comparative analysis of nine LAB genomes in a single study, covering wide application areas varying from dairy fermentations to wine production [70]. Presently over 100 genomes of LAB are deposited in public databases (Fig. 1). The majority of these genomes have not been closed as this is notably difficult because of the presence of multiple repetitive sequences, such as Insertion Sequences (ISs). However, as a framework of around 25 completely closed LAB genomes is presently available, comparative and other detailed analyses offer new leads for functional studies as discussed below.

**Table 3:** Paradigm strains of LAB. A listing of the most relevant LAB strains, their genome size and year of publication is provided. Moreover, the presence of CRISPR sequences is given with a summary of the reported transformation frequency, varying from very high, high, medium to low, representing 10^6^-10^8^, 10^4^-10^6^, 10^2^-10^4^, 10^0^-10^2^ transformants per ug of DNA, respectively. ND, indicates not determined.

Paradigms Strains	Genome Size	Publication Year	Transformation	CRISPR	References
*Lactococcus lactis IL1403*	2.4 Mb	2001	very high	none	[50]
*Lactococcus lactis MG1361*	2.5 Mb	2007	very high	none	[51]
*Lactobacillus plantarum WCFS1*	3.3 Mb	2003	very high	none	[54]
*Lactobacillus acidophilus NCFM*	2.0 Mb	2005	high	none	[63]
*Lactobacillus rhamnosus GG*	3.3 Mb	2009	medium	yes	[31]
*Lactobacillus salivarius* UCC118	2.0 Mb	2006	medium	yes	[64]
*Lactobacillus bulgaricus*	1.9 Mb	2006	low	yes	[65]
*Streptococcus thermophilus*	2.0 Mb	2004	medium	yes	[66]

## Comparative, pan and meta-genomics developments

In silico comparative genomics has been applied ever since the first complete genomes of the same genus were reported and included those of *Lactobacillus plantarum* and *Lactobacillus johnsonii*[71]. However, these genomes differ in size by about 1 Mb and limited conservation was observed [72]. On a larger scale, comparative genomics was applied to explain the origin of LAB based on a set of a dozen different LAB genomes [70]. An evolutionary tree of LAB could be generated that explained the present LAB genomes by a series of multiple gene losses and acquisitions. This important concept also indicated that LAB and *Bacillus subtilis* shared a common ancestor, providing a teleological explanation for the success of many genetic tools that have been developed for *Bacillus* systems and work efficiently in LAB and vice versa. The recent observation that *Streptococcus thermophilus* and possibly other LAB may become competent and hence are naturally transformable provides another practically important example [73]. Natural transformation in conjunction with other mutation selection systems would provide an important tool to expand the genetic potential of LAB without them being labeled as GMO.

Most of the LAB genomes contain around 2000-3000 genes. The question arises how different these genes are and whether a core genome can be found. Comparative analysis of 20 completely sequenced *Lactobacillus* genomes showed the pan genome to contain approximately 14000 genes and indicated the presence of a core genome of 383 orthologous genes [74]. These and other comparative studies confirmed the fact that about one third of the pan genome can not be accurately annotated and that there exists series of wrongly or poorly annotated genes. In silico comparative genomics approaches can address those and together with experimental analyses lead to improved annotations and discovery of new functions. The first of these were performed by comparative genome hybridization using a microarray of *Lactobacillus plantarum* WCFS1 that was tested with DNA of a dozen of related strains from different habitats [75]. The results indicated the presence of specific genomic regions, called life-style islands that were already predicted from the genome with unusual G+C content and varied between the different strains [54]. Moreover, gene-trait matching was performed and this led to the discovery of a mannose-binding protein encoding gene that is present in probiotic strains of *Lactobacillus plantarum* and may contribute to competitive exclusion with pathogenic *Escherichia coli* strains that are known to bind to mannose [76]. Various other comparative genomic studies capitalized on large strain collections and advanced bioinformatic tools developed to allow for rapid gene-trait matching [77]. Recently, this led to the assignment of a series of candidate genes involved in immunological signaling [78]. The advantage of comparative genome hybridization is that use is made of the natural biodiversity of LAB and rapid molecular insight is generated. From an applied perspective this is highly desired as it is a non-GMO approach. However, as all comparisons are realized by using an array of a single strain, no insight in any new coding sequences is generated and this can only be realized by sequence analysis and in silico comparisons.

Advanced comparative genomics and metagenomics approaches can nowadays be realized by deep sequence analysis using Next Generation Technology (NGT) sequencing approaches. The first LAB genome that was completed using NGT sequencing was that of *Lactobacillus rhamnosus* GG and all following ones have been based on some form of NGT sequencing. In addition, deep resequencing has been realized that generated the genome sequence of the well-known strain NZ9000 used as host for the NICE system [44] and this also allowed for correcting sequence errors in the genome of its parent MG1363 [52]. Moreover, draft genome sequences that are almost full length have been generated as is illustrated by the genomic sequencing of a dozen of *Lactobacillus* strains as part of the Human Microbiome Project [79]. The use of NGT sequencing approaches explains the rapid boost in the number of LAB genomes deposited in public databases in recent years [Fig. 1]. To exemplify the rapid analysis, we determined recently draft genomic sequences of approximately 100 strains of *Lactobacillus rhamnosus*-like strains obtained from food, clinical and other environmental samples using NGT approaches [WMdV, unpublished observations].

Next to advanced comparative genomics also metagenomic approaches are being applied using NGT sequencing and these are specifically suitable for the analysis of mixed cultures. The practical importance of mixed strain starters is enormous as cultures of LAB consisting of multiple strains of undefined composition are widely used in the dairy and other food industries. In many cases these mixed strain starters are used intentionally to increase the diversity, and hence resilience and robustness. In other cases, mixed cultures are used unintentionally and relate to the heterogeneity that originates when strains of LAB are repeatedly subcultured [80]. Many mixed cultures also contain bacteriophages that contribute to the equilibrium between the strains, as has been illustrated for the phage–carrying situation [81]. Such mixed starter cultures can be seen as developing ecosystems. Hence, the application of NGT sequencing approaches to describe its collective metagenome will be instrumental in understanding the behavior of these mixed cultures and explaining as well as predicting their success in industrial fermentations.

## High throughput developments – functional analysis and screening

One of the great achievements of the omics revolution is the development of high throughput functional genomics approaches. Notably transcriptomics studies have been instrumental in analyzing the response of LAB to different environments and over 100 papers have been published addressing a variety of stresses, growth conditions and culturing regimens. Among the most important practical discoveries was the finding that *Lactococcus lactis* and other LAB, when provided with the appropriate cofactors, such as heme, could use alternative electron acceptors and hence respire rather than ferment [82]. Following the first description of the use of molecular oxygen by *Lactococcus lactis* MG1363 [83], a variety of studies have followed that have been reviewed recently [84]. Transcriptional studies allowed to identify the genes involved in the use of molecular oxygen that resulted in a wide range of applications, notably faster growth and higher yield of starter cultures [85]. Biochemical studies confirmed that indeed a proton motive force was generated during respiration illustrating the link between transcriptional and functional studies [86]. Moreover, some LAB also were found to use nitrate as terminal electron acceptor, expanding further the possibilities beyond fermentation [87].

Analysis of the transcriptional response is a powerful tool to study fast responses. The analysis of the response to acid stress was the first microarray study reported and related to *Lactobacillus plantarum*[88]. Subsequently, a large set of hundreds of transcriptional responses have been collected that have been systematically addressed by advanced correlation analysis using newly developed tools [89]. This helped to find a series of supergenomic networks and provided further insight in global regulation systems as recently reported [89]. Moreover, the use of metabolic maps based on the genome-based modeling [59] permitted rapid display and analysis of the expressed genes and in such a way it was found that during aerobic growth of *Lactobacillus plantarum*, carbon dioxide was required to allow rapid and uninterrupted growth [90].

Another important observation made by using a community transcriptomics approach was the finding that the human isolate *Lactobacillus plantarum e*xpressed a completely different set of genes in the intestine as compared to growth in laboratory media [57]. This partly explained the large coding capacity of over 3000 genes of this human isolate. Remarkably, a highly comparable expression profile in mice and human was generated, indicating similarities in the intestinal adaptation [57]. Due to the high signal to noise ratio of the microarrays, this analysis could be performed in the complex intestinal ecosystem where LAB represent a fraction of the total number of cells. More advanced metatranscriptomics studies that also capitalize on this property provided by microarrays aim to analyze the transcriptional response in complex food ecosystems. This requires microarrays of multiple strains and the feasibility can be exemplified with the consortium of *Lactobacillus bulgaricus* and *Streptococcus thermophilus* that is involved in yoghurt fermentation [91]. Parallel global transcriptome analysis of the consortium during growth in milk provided evidence for the involvement of specific compounds and metabolic pathways in the interactions between the two strains [91,92]. This is one of the few studies that is performed in an industrial environment and the generated insight may be extrapolated to design stable interactions of other consortia of interacting strains, including probiotics.

It is known from earlier studies that characteristic LAB communities are developing in fermented foods such as cheese [93]. Microarrays representing multiple LAB genomes have been used to determine the response during these successions. Insight in the biodiversity and activity was generated for the fermentation of kimchi, a traditional Korean vegetable product, described to contain various health-promoting factors [94]. Similar approaches were recently applied to complex and uncontrolled sour dough fermentations [95]. These examples all relate to fermentations of plant-derived products and it may be expected that use of these approaches will also be instrumental in analyzing the events in industrial dairy fermentations with complex cultures. Presently, NGT sequencing is being applied to determine global transcriptional responses and this holds great potential as the sequence depth is increasing steadily [96]. These approaches are specifically useful to address small RNAs and their processing, possibly non-coding regions, and LAB communities that have not yet been characterized completely.

Proteomics studies have been instrumental in identifying adaptations that take place over a longer time frame than transcriptomics responses. In a recent systematic study the proteomic and transcriptional responses of the probiotic *Lactobacillus rhamnosus* GG to bile acid were compared [97]. Both approaches were complementary to each other and pointed towards the reduction in EPS production following exposure to bile acid, suggestion that in the intestinal tract cell-envelope located proteins, such as anchored by the action of sortases, are exposed to the extracellular environment. This provided the basis for new probiotic mechanisms to be analyzed *Lactobacillus rhamnosus* GG, such as the exposure of the mucus binding protein or the effect of pili in reducing the IL-8 stimulation by lipotechoic acids in human enterocytes that both are more pronounced in EPS-deficient cells [98].

While analysis of relevant metabolites is standard physiological practice, global metabolomics studies have not been reported frequently for LAB, which may be explained by the complex set of biochemical instruments needed for high throughput metabolic analyses. However, a recent comparative metabolomics and transcriptomics analysis of folate-overproducing *Lactobacillus plantarum* cells was reported to explain its reducing effect on the growth rate [99] . Remarkably, only little effect on the transcriptome and metabolome was observed but the great impact on the growth rate was explained by the gratuitous production of large amounts of folate-related transcripts and proteins.

The usefulness of transcriptomics, proteomics and metabolomics studies depend largely on the stability of the detected molecules. This is not the case with systems that can operate along all time scales and can be detected infinitely as they concern permanent genetic changes. This holds for the in vitro expression technology (IVET) approaches and variations thereof. These are high throughput systems that capitalize on upregulation of gene expression and need efficient transformation systems or intermediary hosts. The first IVET studies in LAB were performed in *Lactobacillus plantarum* and revealed genes that were upregulated in the murine host [100]. These pioneering studies required the optimization of tools that were later used in IVET studies addressing the response of *Lactococcus lactis* cultures in the cheese production process. This is a highly relevant approach and this study revealed a series of genes involved in the cheese maturation process [101]. A great variety of efforts focusing on reducing cheese maturation times have been reported, the most advanced being the use of cell density induced lysis of lactococcal cells [46]. However, there is a great need to extend beyond that knowledge and develop systems that are based on the natural induced genes as have now been exposed using the IVET approach.

In general, omics and the other described high throughput approaches generate leads that form the basis for functional genomics approaches. The throughput of these functional studies is often a bottleneck but has been greatly improved by efficient transformation and expression platforms (see above) as well as systems for generating rapidly multiple mutations in a single strain. An efficient *cre-lox* system for obtaining such multiple mutants in an efficient and successive way have been described for *Lactobacillus plantarum* that may be considered as self-cloning [102]. On the practical side also high throughput developments have been reported and an ingenious system of small cheeses has been designed and used [103]. Finally, a variety of in vitro systems that may predict practical conditions have been reported, such as the use of non-growing cells of *Lactococcus lactis* strains for the rapid analysis of flavor production [104].

To avoid all GMO related issues, the best optimization of industrial LAB is to generate mutants. While the use of advanced genetic systems, such as mutator strains, has been described [105], natural means of creating diversity are also feasible and may be sufficiently frequent to generate variation that subsequently can be selected. This has been shown for the adaptive evolution of *Lactococcus lactis* for over 1000 generations that revealed the mobility and subsequent mutation by IS elements [106]. It is well known that IS elements are a powerful source of generating variation as has been illustrated for the construction of lactose-deficient mutants to prevent post-fermentation acidification in *Lactobacillus bulgaricus*[107]. Another dimension has been generated by all kinds of high throughput equipment and is supported by whole genome NGT resequencing to provide insight in the nature of the generated mutations. This has been tested in the adaptive evolution of yoghurt strains that had not previously been grown together in a consortium. It was found that more than 1000 generations of growth resulted in stable consortia of naïve strains of *Lactobacillus bulgaricus* and *Streptococcus thermophilus* that even outperformed industrial consortia for growth rate [92]. Whole genome re-sequencing detected multiple mutations in both species that affected metabolic pathways relating to their interdependence. These results illustrate the need for an advanced understanding of the metabolic network relations that can be exploited to generate stable consortia of LAB consisting of fermentative or probiotic strains.

## Systems and synthetic biology – integrating metabolism, genetics & application

To capture the full potential of the omics and other high throughput development, a systems biology approach is essential [108]. Basically, this allows incorporating experimental data into an intellectual framework of a model and hence integrate these into a hypothesis generating system. In the full circle of the systems biology, the generated hypothesis can be tested experimentally, optimizing and extending the models. The power of this approach can be illustrated by examples of the advances made in *Lactobacillus plantarum* as reviewed recently [60]. Based on the *Lactobacillus plantarum* genome, a metabolic model was created that described very well the growth and product formation when grown in minimal media on glucose [59]. What the flux balance analysis model also predicted was that *Lactobacillus plantarum* strain not only should grow efficiently on hexose sugars but also on glycerol, a C3 compound. However, the wild-type strain showed only a very low growth rate on glycerol, indicating the presence of an unexpected bottleneck. By adaptive evolution supported by efficient growth using oxygen as electron acceptor, a derivative strain of *Lactobacillus plantarum* was obtained that completely converted glycerol into mainly lactic acid as predicted by the genome-based model [109]. In this systems biology approach, the experimental data provided further insight as genomic resequencing of the resultant strain showed promoter mutations and relieve of catabolite repression of the glycerol operon [E.J. Smid, personal communication]. The inability to predict this bottleneck among others results from the absence of hierarchal control data in the metabolic model and their incorporation would provide the next level of sophistication.

It is evident that systems biology approaches capitalize on genomic information, genome based modeling and high throughput experimentation. As it aims to generate productive outcomes, as such it integrates genetics, metabolism and applications. Moreover, systems biology can capture previous knowledge and this even increases its power [108]. Hence, the paradigm LAB are ideal organisms to further exploit using systems biology approaches. Notably for *Lactococcus lactis* a large body of information has been collected, including a variety of kinetic, static and genome-based models that have been reviewed [109,110]. However, new and refined models are continuously emerging testifying for the interest in the systems biology approach and this paradigm LAB strain [112-114]. Moreover, a wealth of metabolic data is available for *Lactococcus lactis* MG1363 as it has been used in a multitude of metabolic engineering experiments [115]. Many of these have been highly successful and these are summarized here since under optimal conditions the flux distribution reaches the theoretical maximum (Table 4). These extreme fluxes are highly unusual and affirm the usefulness of *Lactococcus lactis* as a host for metabolic engineering in which growth and production can be uncoupled [115]. Various factors may contribute to this, including its simple metabolism, limited redundancy and few high level control systems in its small genome. Its relative simplicity was confirmed by comparative modeling studies where genome-based models and transcriptional responses of *Lactococus lactis*, *Lactobacillus plantarum* and *Streptococus thermophilus* were compared [119]. A last level of sophistication in the modeling approach is the construction of models for mixed cultures. This has been realized for the yoghurt consortium consisting of *Streptococcus thermophilus* and *Lactobacillus bulgaricus* and provided important support for the explanation of experimental data [92]. Further modeling of more complex consortia of LAB such as in mixed cultures is also feasible and bottom-up as well as top-down approaches to realize this have been recently reviewed [120].

The unusual success of metabolic engineering approaches in *Lactococcus lactis* [Table 4] together with its high transformation efficiency [Table 2] indicates that this and possibly other LAB are promising candidates for synthetic biology applications. Synthetic DNA was rapidly incorporated in the genetic engineering of LAB and was already 20 years ago used to optimize constructs from *Lactococcus lactis* expression [121]. In addition, some of the first synthetic promoters were designed for *Lactococcus lactis*[122]. However, the present developments in synthetic biology not only allow gene fragments, cassettes or operons to be synthesized but even complete genomes [123]. While there are many bottlenecks, varying from modeling multiple gene functions to booting up new genomes, synthetic biology is the ultimate engineering approach that capitalizes upon a real biological understanding. By exploiting designed DNA up to the size of a complete genome, synthetic biology builds upon the systems biology approaches that have been discussed above.

What synthetic biology approaches could be applied to LAB ? One avenue is to build upon functions that are present and can be optimized, given the profound knowledge of their biology. This can include the production of high value ingredients such as vitamin B12 produced by *Lactobacillus reuteri*[124], specific flavors from amino acids as synthesized by *Lactococcus lactis* or *Streptococcus thermophilus*[119], or the production of plant-stimulating compounds that can be produced by *Lactobacillus plantarum* at zero-growth conditions [125]. Similarly, one can think of new anti-infectives based on the large potential of LAB in the production of bacteriocins or lantibiotics, such as nisin [127]. However, these are all products that are rather traditional. More exciting are new products or strains. One possibility is to generate biobricks of probiotic gene functions [[128]; Table 2] that can be incorporated in different hosts and, in varying combinations, tested in high throughput systems for functionality. Similarly, new LAB vaccine strains could be developed where large synthetic DNA fragments are employed. Significant developments have emerged since the first description of lactoccci as oral vaccines [128,62] and specific helper functions that boost the antigenic response have recently been discovered [129]. A final option is to further capitalize on the physiological strengths of LAB. These include their high stress resistance, tolerance to low pH, and uncoupling of growth and production. This can be linked to high growth rates, sometimes at elevated temperatures, and the use of both hexoses and C5 sugars, while redox balancing can be realized in several ways by respiration [82,84] or the use of water-forming NADH oxidases as demonstrated previously [130]. There are great opportunities, including the production of organic acids other than lactic acid, such as succinic acid, malic acid or propionic acid. Moreover, many LAB have a high level of alcohol tolerance and are even observed as contaminants of commercial alcohol production [131]. Hence, the possibilities of producing butanol, isobutanol or higher alcohols can be considered and several engineering efforts towards generating these products have already been reported [132,133]. However, in order to be competitive with present white biotechnology production systems, the LAB hosts should be optimized for prototrophic growth on simple media obviating the need for yeast extract or other additions. With the present metabolic models in combination with synthetic biology approaches this should be feasible and then we would be entering into a new era where exciting new developments are to be expected.

**Table 4:** Selected metabolic engineering studies with *Lactococcus lactis* MG1363. The new product, its properties and the efficiency of the flux redistribution from glucose

Product	Functional Properties	Flux	Reference
Alanine	Flavor, L-Amino Acid	> 99	[116]
α-Acetolactate	Flavor, Precursor	~ 70	[117]
Aceetaldehyde	Flavor, Conservation	~ 50	[118]

## Conclusions

The genomic characterization of LAB has rocketed and presently over 25 complete and 100 or so nearly complete genomes are available. Many of these derive from strains that serve as scientific paradigms and have reached the market place as the industrial use of LAB is worth over a 100 Billion Euro per year. Several paradigm LAB have been presented here with their most salient features that include genome based modeling as well as systems and synthetic biology approaches. The field of LAB is developing really rapidly and an impressive set of discoveries have been made that had not been anticipated ten years ago [134]. When considering the large amount of knowledge available, the logic step is to further improve existing products and start developing NGT products for food, pharma and white biotechnology. It is an expectation and desire that this review contributes to these exciting new developments by inspiring new scientific talents to do so.

## Competing interests

The author declares that he has no competing interests.

## List of abbreviations used

LAB: Lactic Acid Bacteria; GMO: Genetically Modified Organism; IVET: In Vitro Expression Technology; NGT: Next Generation Technology; NICE: Nisin Induced Controlled Expression; SLP: Surface Layer Protein; LTA: Lipotechoic Acid.

This article has been published as part of *Microbial Cell Factories* Volume 10 Supplement 1, 2011: Proceedings of the 10th Symposium on Lactic Acid Bacterium. The full contents of the supplement are available online at http://www.microbialcellfactories.com/supplements/10/S1.
